# Linear and conformational determinants of visceral leishmaniasis diagnostic antigens rK28 and rK39

**DOI:** 10.1186/s13071-022-05495-1

**Published:** 2022-10-22

**Authors:** Poppy Simonson, Tapan Bhattacharyya, Sayda El-Safi, Michael A. Miles

**Affiliations:** 1 Faculty of Infectious and Tropical Diseases, London School of Hygiene and Tropical Medicine, London, UK; 2 Faculty of Medicine, University of Khartoum, Khartoum, Sudan

**Keywords:** Visceral leishmaniasis, Serology, Antigens, rK39/rK28, Conformation, Linear epitopes, Specificity

## Abstract

**Background:**

Recombinant antigens rK39 (based on kinesin sequence) and rK28 (comprising kinesin and HASPB sequences) are a mainstay of serological diagnosis for visceral leishmaniasis (VL). However, their key epitopes and the significance of their structural conformation are not clearly defined, particularly in relation to reported cross-reactivity with sera from patients with malaria, schistosomiasis, and tuberculosis.

**Methods:**

To assess the effect of conformation on antigenicity with Sudanese VL sera, antigens rK39 and rK28 were heat-denatured at 95 °C for 10 min and then assayed by enzyme-linked immunosorbent assay (ELISA). Amino acid sequences of rK39 and rK28 were submitted to NCBI BLASTp to assess homology with *Plasmodium*, *Schistosoma*, and *Mycobacterium*.

**Results:**

Heat denaturation significantly diminished the antigenicity of rK39 compared to non-denatured antigen (*P* = 0.001), but not for rK28 (*P* = 0.275). In BLASTp searches, HASPB sequences from rK28 had similarities with sequences from *Plasmodium*, encompassing software-predicted B-cell epitopes.

**Conclusions:**

The antigenicity of rK39 appears to be dependent on structural conformation, whereas that of rK28 depends on linear sequence. HASPB sequence homology with *Plasmodium* may be responsible for the reported cross-reactivity of rK28 with malaria sera. Further work is warranted to refine the specificity of these antigens.

**Graphical Abstract:**

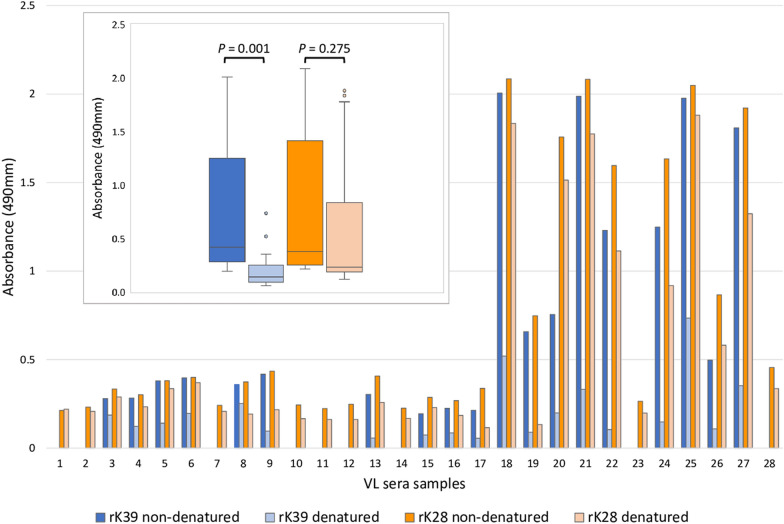

## Background

Visceral leishmaniasis (VL) is a protozoal disease spread by phlebotomine sand flies, caused by species of the *Leishmania* (*Leishmania*) *donovani* complex: anthroponotic *L.* (*L.*) *donovani* principally in South Asia and East Africa; and zoonotic *L.* (*L.*) *infantum* in Southern Europe, North Africa, West and Central Asia, and Central and South America [1, 2]. Most cases of symptomatic VL are fatal without treatment, and it is a significant cause of worldwide mortality and morbidity [3]. As a neglected tropical disease (NTD), VL lacks safe effective treatments and good-quality diagnostic tests. This hampers disease control efforts for humans and, in the case of *L. infantum* VL, for the canine reservoir. Serological diagnostic methods, such as enzyme-linked immunosorbent assay (ELISA) and rapid diagnostic tests (RDTs) adapted to low-resource settings, are limited by their inability to differentiate between prior exposure and active infection, and by susceptibility to cross-reactivity with other infections [1, 4, 5]. Molecular methods such as polymerase chain reaction (PCR) and recently loop-mediated isothermal amplification (LAMP) require training and technology, which is a barrier to their use in the frequently resource-limited settings where VL is endemic [6–8].

The recombinant antigen rK39, comprising a 46-amino acid region followed by 6.5 non-identical repeats of 39-amino acids of a kinesin-like protein, has been a mainstay of serological diagnosis for VL for decades since its identification from Brazilian *L. infantum* (synonym *L. chagasi*) [9]. RDTs using rK39 have been found to have lower sensitivity in East Africa than the Indian subcontinent [10–12]. One reason for this may be the significant diversity which has been found in amino acid sequences of rK39 homologues of East African *L. donovani* strains, compared to that of the *L. infantum*-derived diagnostic antigen rK39, and to South Asian homologues [13]. The repeat regions within rK39 are themselves non-identical [6, 9].

Pattabhi et al. [14] reported the synthetic fusion protein rK28, containing kinesin repeats from Sudanese *L. donovani* flanked by sequences of the hydrophilic acylated surface proteins (HASPB) from an Ethiopian *L. donovani*, to improve sensitivity. The HASPB1 and HASPB2 sequences in rK28 have also been known as K26 and K9, respectively [15]. There have been reports of cross-reactivity with rK28 and sera from patients infected with malaria, tuberculosis, and schistosomiasis [4, 16]. Variation in the sequence and distribution of HASPB sequences has also been reported, including in East Africa [13, 17, 18].

The crucial epitopes of rK39 and rK28 have not been well characterized, and they may reside not only in the primary sequence of the amino acids but also in the structural conformation of these antigens. Here, we assess the effect of conformational denaturation on their antigenicity with sera from Sudanese VL patients.

## Methods

### Ethics statement

Sudanese VL serum samples were collected in 2011–2012 from Gedaref state, Sudan, as part of the NIDIAG research consortium (https://cordis.europa.eu/project/rcn/97322_en.html). Approval was by the Ethical Research Committee, the Medical and Health Sciences Campus, University of Khartoum, the National Health Research Ethics Committee, Federal Ministry of Health, Sudan, and by the London School of Hygiene and Tropical Medicine Ethics Committee, United Kingdom. Written informed consent was obtained from adult subjects or from the parents or guardians of individuals less than 18 years of age (who also gave verbal consent).

### VL serum samples

Cases of VL had been diagnosed by microscopy of bone marrow and/or lymph node aspirates, in conjunction with serological assays in some cases. A total of *n* = 42 VL samples were assayed; of these, included in the final analysis (where reactivity against the non-denatured antigen was above the cut-off value) were *n* = 19 for rK39 and *n* = 27 for rK28. Sera from endemic healthy controls (healthy Sudanese people who were seronegative against VL antigens; *n* = 20) were used as negative control.

### ELISA

#### Recombinant antigens

rK28 (CTK Biotech, USA) and rK39 (RAG0061, Rekom Biotech, Spain) were obtained commercially. To compare the antigenicity of heat-denatured and non-denatured rK39 and rK28, these antigens were first diluted in coating buffer (15 mM Na_2_CO_3_, 34 mM NaHCO_3_, pH 9.6) at approximately 0.25 μg/ml. A portion of each of these antigen solutions was then heat-denatured at 95 °C for 10 min in a hot block, whereas the remainder was not denatured. The ELISA plate (735-0465, VWR, UK) was divided into quadrants, coated with 100 μl/well as follows: top-left quadrant, non-denatured rK39; top right, rK39 heat-denatured; bottom left, non-denatured rK28; bottom right, rK28 heat-denatured. Following overnight incubation at 4 °C, wells were washed three times with phosphate-buffered saline (PBS)/0.05% Tween 20 (PBST); then 200 μl/well of blocking buffer (PBS/2% skimmed milk powder; Premier Foods, UK) was applied to the whole plate and incubated for 2 h at 37 °C. Following three washes, 100 μl/well of serum diluted 1:100 in PBST/2% milk was added, such that these samples were arranged identically in each quadrant. Following incubation at 37 °C for 1 h and six washes in PBST, 100 μl/well of 1:1000 dilution in PBST/2% milk of horseradish peroxidase-labelled anti-human IgG1 (ab99774, Abcam, UK) was added to the whole plate. We have previously shown the potential of immunoglobulin G1 (IgG1) for assessing treatment outcome [19, 20]; thus we were interested here in applying IgG1 detection in the current assay context, rather than IgG.

Following incubation at 37 °C for 1 h and 6 PBST washes, 100 μl/well of substrate solution (50 mM phosphate/citrate buffer, pH 5.0) containing 2 mM σ-phenylenediamine HCl (P1526, Sigma Aldrich) and 0.009% H_2_O_2_ (216763, Sigma Aldrich) was added to the entire plate and incubated in the dark. Reactions were stopped by the addition of 50 μl/well of 2 M H_2_SO_4_, and absorbance was read at 490 nm. Samples were assayed on duplicate plates simultaneously. Each quadrant contained positive and negative (endemic healthy) controls.

We did not use beta-mercaptoethanol in the denaturation, as each of the rK28 and rK39 recombinant protein sequences have only one Cys residue, so disulphide bonds are not likely to be present; we did not treat the antigens with sodium dodecyl sulphate, to avoid any issues with subsequent coating of the ELISA plate.

#### Statistical analysis

The cut–off value was calculated from the mean plus three standard deviations of the endemic healthy control sera. Only those samples for which the non-denatured antigen (rK39 or rK28) had a reading above the cut-off were included in subsequent analysis. *P*-values were determined by an unpaired two-sample *t*-test, assuming equal variance, using Microsoft Excel.

#### Bioinformatics

We wished to assess the level of homology between the linear sequences of the VL antigens and potential cross-reacting species. Thus, the HASPB1, HASPB2, and kinesin amino acid sequences of rK28 (GenBank HM594686) and the 46-amino acid non-repeat region and 6.5 × 39-amino acid repeats of rK39 (GenBank L07879) were submitted to BLASTp searches (https://blast.ncbi.nlm.nih.gov), both unrestricted by taxon and restricted to *Schistosoma*, *Plasmodium*, and *Mycobacterium*.

We used BepiPred-2.0 (https://services.healthtech.dtu.dk/service.php?BepiPred-2.0) to predict linear B-cell epitopes in the HASPB1 sequence of rK28 (GenBank HM594686).

## Results

### Denaturation diminishes the antigenicity of rK39 to a greater extent than for rK28

**Fig. 1:**
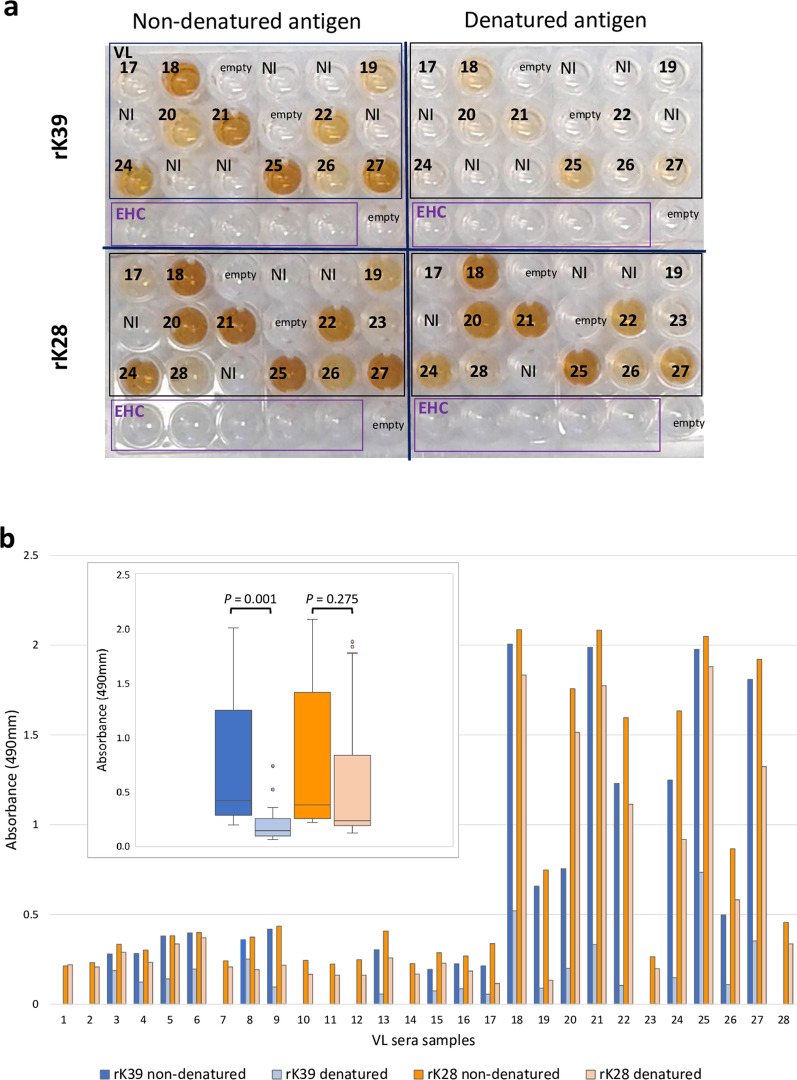
Denaturation diminishes the antigenicity of rK39 to a greater extent than for rK28. **a** Example of ELISA plate with coating of antigens rK39 and rK28 non-denatured or denatured; in each quadrant, the same serum samples are arranged identically. *VL* Sudanese visceral leishmaniasis, *EHC* endemic healthy control, *NI*, samples not included in subsequent analysis, as the reaction with non-denatured antigen was below cut-off. **b** Mean absorbance values of individual VL sera are shown, against antigens non-denatured (dark bars) or heat-denatured (corresponding light bars); cut-off value is normalized as *y*-axis zero. Inset: box-and-whisker plot of composite absorbance values of VL sera against denatured and non-denatured rK28 and rK39

Sudanese VL sera were reacted with heat-denatured or non-denatured recombinant antigens rK39 and rK28, these four antigen formats being arranged in quadrants of the same ELISA plate (Fig. 1a). The most striking observation was that the antigenicity of heat-denatured rK39 was greatly diminished when compared with non-denatured. We considered first whether samples gave a positive response above the cut-off value with non-denatured antigen; samples at or below the cut-off were excluded from the subsequent analysis. When considered as a group, heat denaturation significantly diminished the antigenicity of rK39 (*P* = 0.001), whilst that of rK28 was not significantly altered (*P* = 0.275) (Fig. 1b inset).

### Bioinformatics analysis reveals HASPB correspondence with *Plasmodium* sequences

As rK28 has previously been reported to give cross-reactions with sera from patients with malaria, pulmonary tuberculosis, and intestinal schistosomiasis, we used the amino acid sequences of the HASPB1, kinesin, and HASPB2 components of this fusion protein antigen in BLASTp searches against these pathogens.

**Fig. 2:**
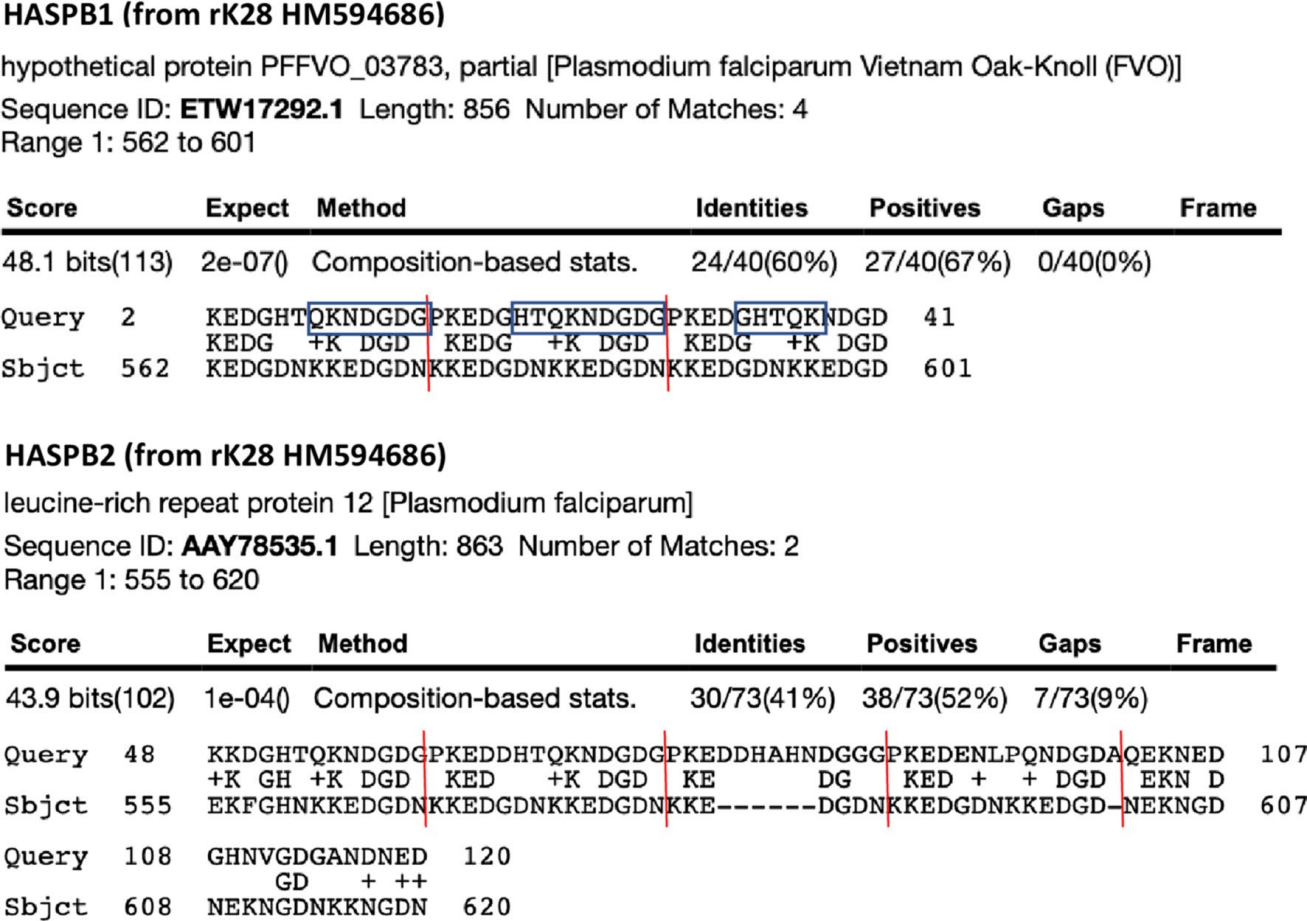
BLASTp searches identify HASPB sequence similarity in *Plasmodium*. The matches with HASPB sequences principally involved the repeats containing the central His-Thr-Gln-Lys-Asn (HTQKN) sequence. The different repeats are delineated by the red lines. Query: rK28 HASPB sequences derived from GenBank HM594686. Sbjct: retrieved sequence match. + symbol: amino acids with similar physicochemical properties. Blue boxes: predicted HASPB1 epitope sequences

Apart from expected similarities to *Leishmania*, searches retrieved matches of HASPB sequences to *Plasmodium*. In the example shown in Fig. 2, the matches with the *Plasmodium* leucine-rich repeat protein 12 (GenBank AAY78535.1) involved the central His-Thr-Gln-Lys-Asn sequence type of HASPB, but not the alternative central His-Ala-His-Asn sequence type. No significant similarity was found between HASPB and sequences from *Schistosoma* or *Mycobacterium*. Kinesin sequences from rK28 did not find significant similarity with these non-*Leishmania* genera.

Prediction software (with 0.65 threshold applied) indicated that the HASPB1 epitope resides in regions with high homology to *Plasmodium* (Fig. 2, upper panel).

BLASTp searches using the rK39 sequence did not retrieve matches with *Plasmodium*, *Schistosoma*, or *Mycobacterium.*

## Discussion

Here we report that the antigenicity of rK39, which is composed almost entirely of kinesin 39-amino acid repeats, is dependent on conformation, not linear sequence. Gerald et al. [21] reported a software-predicted coiled-coil structure of kinesin repeats in a Sudanese *L. donovani* strain; such a conformation would be highly susceptible to denaturation, disrupting conformational epitopes.

In contrast, rK28 antigenicity was only slightly decreased following heat denaturation. As the kinesin component forms a much smaller proportion of the entire rK28 antigen than in rK39, we infer that any loss of kinesin antigenicity due to conformation is mitigated by the presence of the flanking HASPB1 and HASPB2 sequences, which thus indicates dependence not on their conformation, but (linear) amino acid sequence.

Archived Sudanese VL sera were used here due to availability. In the future, assessing the antigenicity of heat-denatured rK28 and rK39 with VL sera from other regions is of interest, given differing local profiles of *Leishmania* species and strains, and of co-infections which may cross-react. We did not use a no-antigen control on our ELISA plates; however, all the endemic healthy controls were negative with rK28 and rK39 in denatured and non-denatured formats.

We were primarily interested in IgG1 detection, due to its potential in monitoring treatment outcome: we found in previous studies using Indian VL samples that cure is associated with decreased IgG1 levels [19, 20]. Higher seropositivity rates may occur with IgG ELISA and non-denatured antigens, and we do not propose IgG1 as a first-line diagnostic. Further work may be of interest to investigate whether these results are applicable to other IgG subclasses.

BLASTp searches revealed correspondence between the HASPB1 used in rK28 and *Plasmodium* sequences. Bezuneh et al. [4] and Kassa et al. [16] reported cross-reactivity of rK28 in East Africa, with sera from malaria, tuberculosis, and schistosome infections, although previous exposure to VL could not be excluded by those authors. Our finding that linear sequence is crucial for rK28 antigenicity, residing in the HASPB components of this fusion protein, is of great interest for understanding BLASTp matches and co-endemicity of other diseases in VL-endemic areas. Future studies using overlapping HASPB peptides may refine these epitopes. Though rK28 has not yet been used in a commercial RDT for VL diagnosis in humans, an rK28-based RDT is widely used for canine VL and shows high sensitivity and specificity [22, 23]. Whilst some authors report cross-reactivity between rK28 and canine pathogens such as *Babesia* spp. [24], high sensitivity of rK28 serology of dogs with *L. infantum* VL in northern Argentina was not found to be related to cross-reactivity [8].

In *Leishmania major*, HASPB has an essential role during parasite development in the phlebotomine vector [25]. Although this protein is expressed on the surface and recognized by antibodies, its role in the human host is not clear. As previously reported [13, 17, 18], HASPB polymorphism in East African *L. donovani* strains (including in unexpected PCR amplicons) is found not only as various amino acid changes, but also in the combination of core His-Thr-Gln-Lys-Asn or His-Ala-His-Asn sequences across the repeat region of the antigens. These sequences were not consistently present in the corresponding repeats across strains. The His-Thr-Gln-Lys-Asn sequence is the predominant repeat in rK28 (GenBank HM594686), being present five times compared to a single instance of the His-Ala-His-Asn. A recent human therapeutic vaccine candidate for patients in Sudan with post-kala-alar dermal leishmaniasis, a sequel of VL, used an adenoviral fusion vaccine incorporating this HASPB sequence diversity [26]. Our finding here of the BLASTp match to *Plasmodium* being due to the His-Thr-Gln-Lys-Asn sequence may therefore be of relevance to the future design of diagnostic and vaccine candidates.

Further refinement of these antigens may include elimination of problematic HASPB regions from rK28, to remove *Plasmodium* cross-reactivity but retain antigenicity, and modification of rK39 to improve range of sensitivity, yet retain specificity and accommodate the kinesin sequence diversity of non-identical repeats.

## Conclusions

The antigenicity of rK39 is dependent on structural conformation, whereas that of rK28 depends partially on linear sequence. HASPB sequence homology with *Plasmodium* may be responsible for the reported cross-reactivity of rK28 with malaria sera. Further work is warranted to refine the specificity of these antigens.

## Data Availability

The datasets used and/or analysed during the current study are available from the corresponding author on reasonable request.

## Abbreviations

HASPB: Hydrophilic acylated surface protein B
VL: Visceral leishmaniasis
